# Comparison of maternal and neonatal outcomes between acute fatty liver of pregnancy and hemolysis, elevated liver enzymes and low platelets syndrome: a retrospective cohort study

**DOI:** 10.1186/s12884-021-03761-1

**Published:** 2021-04-12

**Authors:** Sau Xiong Ang, Chie-Pein Chen, Fang-Ju Sun, Chen-Yu Chen

**Affiliations:** 1 Department of Obstetrics and Gynecology, Mackay Memorial Hospital, No. 92, Section 2, Zhong-Shan North Road, 10449 Taipei, Taiwan; 2 Department of Medical Research, Mackay Memorial Hospital, Taipei, Taiwan; 3 Department of Medicine, Mackay Medical College, New Taipei City, Taiwan

**Keywords:** Acute fatty liver of pregnancy, HELLP syndrome, Multiple organ dysfunction, Neonatal morbidity

## Abstract

**Background:**

Acute fatty liver of pregnancy (AFLP) and hemolysis, elevated liver enzymes and low platelets (HELLP) syndrome are two uncommon disorders that mimic each other clinically, but are distinct pathophysiologically. This study aimed to compare maternal and neonatal outcomes between AFLP and HELLP syndrome.

**Methods:**

This retrospective cohort study was performed at a tertiary referral center in Taiwan between June 2004 and April 2020. We used the Swansea Criteria to diagnose AFLP, and the Tennessee Classification System to diagnose HELLP syndrome. Maternal characteristics, laboratory data, complications, and neonatal outcomes were compared. We analyzed the categorical variables with Chi-square test or Fisher’s exact test and continuous variables with Student’s t test or Mann-Whitney U test. Subsequent logistic regression analyses adjusting by potential confounding factors with significant difference were analyzed.

**Results:**

During the study period, 21 women had AFLP and 80 women had HELLP syndrome. There was a higher rate of preeclampsia (95.0 % versus 23.8 %) in the HELLP syndrome group compared to the AFLP group. However, the AFLP group had more other maternal complications including jaundice (85.7 % versus 13.8 %), acute kidney injury (61.9 % versus 15.0 %), disseminated intravascular coagulopathy (66.7 % versus 8.8 %), and sepsis (47.6 % versus 10.0 %) compared to the HELLP syndrome group. Nevertheless, higher rates of small for gestational age neonates (57.1 % versus 33.3 %), neonatal respiratory distress syndrome (39.2 % versus 8.3 %) and neonatal sepsis (34.2 % versus 12.5 %) were noted in the HELLP syndrome group.

**Conclusions:**

AFLP is associated with a higher rate of multiple organ dysfunction in mothers, whereas HELLP syndrome is associated with a higher rate of neonatal morbidity.

## Background

Liver diseases during pregnancy occur in up to 3 % of all pregnant women, and severe forms can cause maternal and perinatal morbidity and mortality [1]. They can be categorized into pregnancy-related and pre-existing liver disorders, of which the former includes hyperemesis gravidarum, intrahepatic cholestasis of pregnancy, preeclampsia with or without severe features, acute fatty liver of pregnancy (AFLP), and hemolysis, elevated liver enzymes and low platelets (HELLP) syndrome [1].

AFLP and HELLP syndrome, two uncommon disorders that mimic each other clinically but are distinct pathophysiologically, often arise during the third trimester and contribute to half of acute liver failure during pregnancy [2]. AFLP occurs in 1:7000-15,000 of pregnancies, compared to an incidence of 0.2–0.8 % for HELLP syndrome [3, 4]. AFLP, defined as the microvesicular fatty infiltration of hepatocytes during pregnancy, was first described by Stander and Cadden [5] in 1934. HELLP syndrome is the combination of hemolysis with microangiopathic blood smears, increased liver enzymes and low platelet counts, and was first termed by Weinstein [6] in 1982. Although still unclear, most previous studies have indicated that the main causes of AFLP are deficiency of fetal long-chain 3-hydroxyacyl coenzyme A dehydrogenase (LCHAD) and defects in fatty acid metabolism during pregnancy [7]. The cause of HELLP syndrome is thought to be due to abnormal placentation and the subsequent release of factors resulting in placental hypoperfusion, ischemia and systemic microangiopathies [8]. It might represent a severe subset of preeclampsia in the spectrum of gestational hypertensive disorders, although 15–20 % of patients diagnosed as having HELLP syndrome are normotensive [9].

AFLP and HELLP syndrome should be managed promptly, yet differentiating these two disorders in timely fashion is difficult as they share similar initial clinical presentations and mainly unspecified gastrointestinal discomfort. Most previous studies were review articles that reported maternal conditions of the two disorders individually [10–12], and few studies have compared the two disorders simultaneously in a cohort study [13, 14]. In our experience, the differential diagnosis between AFLP and HELLP syndrome has not been clearly recognized, we conducted this study to compare the maternal and neonatal outcomes between AFLP and HELLP syndrome, and further discussed the two pregnancy-related liver disorders with regards to their clinical features and laboratory findings.

## Methods

This retrospective cohort study of pregnant women with liver disorders was performed at Mackay Memorial Hospital, a tertiary referral center in Taiwan, from June 2004 to April 2020. Detailed data were collected from obstetric records and neonatal databases using International Classification of Diseases (the ninth and tenth revisions) diagnosis codes. This study was approved by Mackay Memorial Hospital Institutional Review Board (IRB no. 19MMHIS291e), which advised that formal ethical approval with informed consent was unnecessary, as this study constituted a retrospective study. All personal identifiers were anonymized prior to analysis.

Patients with viral hepatitis, autoimmune hepatitis, cholestasis of pregnancy, biliary tract disease and ischemic hepatitis due to postpartum hemorrhage were excluded. We used the Swansea Criteria [3] to diagnose AFLP, and the Tennessee Classification System [15] to diagnose HELLP syndrome (Table 1). AFLP was diagnosed if the patient met six or more of the features in the absence of another explanation. Complete HELLP syndrome was diagnosed if the patient met all of the three laboratory criteria in the absence of another explanation. Patients with one or two of these laboratory criteria were defined as having partial HELLP syndrome and were also included in the study.

**Table 1:** The Swansea Criteria for diagnosis of AFLP and the Tennessee Classification System for diagnosis of HELLP syndrome

Swansea Criteria	Tennessee Classification System
Vomiting	AST > 70 IU/L
Abdominal pain	LDH > 600 IU/L
Polydipsia or polyuria	Platelet < 100,000/µL
Encephalopathy	
Leukocytosis (> 11 × 10^6^/mL)	
Hypoglycemia (< 72 mg/dL)	
Hyperbilirubinemia (> 0.8 mg/dL)	
Impaired liver function (AST or ALT > 42 IU/L)	
Hyperuricemia (> 5.7 mg/dL)	
Hyperammonemia (> 66 µg/dL)	
Impaired renal function (creatinine > 1.7 mg/dL)	
Coagulopathy (PT > 14 s or aPTT > 34 s)	
Ascites or bright liver on ultrasound scan	
Microvesicular steatosis on liver biopsy	

*AFLP* acute fatty liver of pregnancy; *HELLP* hemolysis, elevated liver enzymes and low platelets; *AST* aspartate transaminase; *ALT* alanine aminotransferase; *PT* prothrombin time; *aPTT* activated partial thromboplastin time; *LDH* lactate dehydrogenase

Maternal characteristics, laboratory data, complications, and neonatal outcomes between the two disorders were compared. Chronic hypertension during pregnancy was diagnosed if hypertension (systolic blood pressure ≥ 140 mmHg and/or diastolic blood pressure ≥ 90 mmHg) was first detected at < 20 gestational weeks. Gestational hypertension was diagnosed if new-onset hypertension was detected at ≥ 20 gestational weeks without the presence of proteinuria. Preeclampsia was defined as gestational hypertension combined with proteinuria or new signs of end-organ dysfunction [16]. Preeclampsia with severe features included systolic blood pressure ≥ 160 mmHg or diastolic blood pressure ≥ 110 mmHg, platelet count < 100,000/µL, aspartate transaminase (AST) or alanine aminotransferase (ALT) levels twice the normal concentration, creatinine > 1.1 mg/dL or twice the normal concentration, pulmonary edema, or the new-onset of cerebral or visual disturbances [16]. Pulmonary edema was diagnosed according to clinical and radiological findings. Acute kidney injury (AKI) was defined as an increase in serum creatinine (1.5 times the normal baseline within 7 days or ≥ 0.3 mg/dL within 2 days) or oliguria (urine output < 0.5 mL/kg per hour for 6 h) [17]. Disseminated intravascular coagulopathy (DIC) was diagnosed if the women met three or more of the following criteria: thrombocytopenia (< 100,000/µL), hypofibrinogenemia (< 300 mg/dL), positive D-dimer level (> 40 µg/dL), and prolonged prothrombin time (> 14 s) and activated partial thromboplastin time (> 40 s) [18]. Postpartum hemorrhage was defined as a blood loss of more than 500 mL within 24 h after delivery [19]. Sepsis was diagnosed as infection with an acute increase of ≥ 2 Sequential Organ Failure Assessment points [20]. Small for gestational age (SGA) was defined as a birth weight < 10th percentile, based on the national singleton birth weight percentiles in Taiwan [21]. Intracranial hemorrhage was diagnosed by cranial ultrasound. Respiratory distress syndrome (RDS) and transient tachypnea of the newborn were differentiated by chest image, clinical presentation, and the use of surfactant therapy. Persistent pulmonary hypertension of the newborn was diagnosed by evaluating pre- and post-ductal oxygen saturation, chest image, arterial blood gas, and echocardiography [22].

Expeditious delivery was suggested once AFLP was diagnosed, whereas it was suggested if HELLP syndrome developed at ≥ 34 gestational weeks or earlier if there was maternal distress (such as AKI, DIC, eclampsia, and suspected placental abruption), rapidly worsening laboratory values, uncontrolled hypertension, or non-reassuring fetal conditions [15]. A single course of antenatal betamethasone was administered to the women between 23 and 34 weeks of pregnancy to reduce neonatal RDS if the maternal condition allowed, followed by delivery at 24 to 48 h later.

SPSS version 24.0 (IBM Corporation, Armonk, NY, USA) was used for statistical analyses. The Chi-square test was used for categorical variables, and Fisher’s exact test was used instead when the cell had an expected frequency less than 5. For continuous variables, we applied the Kolmogorov-Smirnov test to determine if variables were normally distributed. The Student’s t test was used for normally distributed data; otherwise, the Mann-Whitney U test was used. Logistic regression analyses adjusting by potential confounding factors with significant difference were performed and results were presented as adjusted B or adjusted odds ratio with 95 % confidence interval in continuous variables and categorical variables respectively. In addition, regression analysis with an S-curve of birth weight against gestational age at delivery was plotted between the AFLP and HELLP syndrome groups. A *P* value < 0.05 was considered to be statistically significant.

## Results

**Fig. 1:**
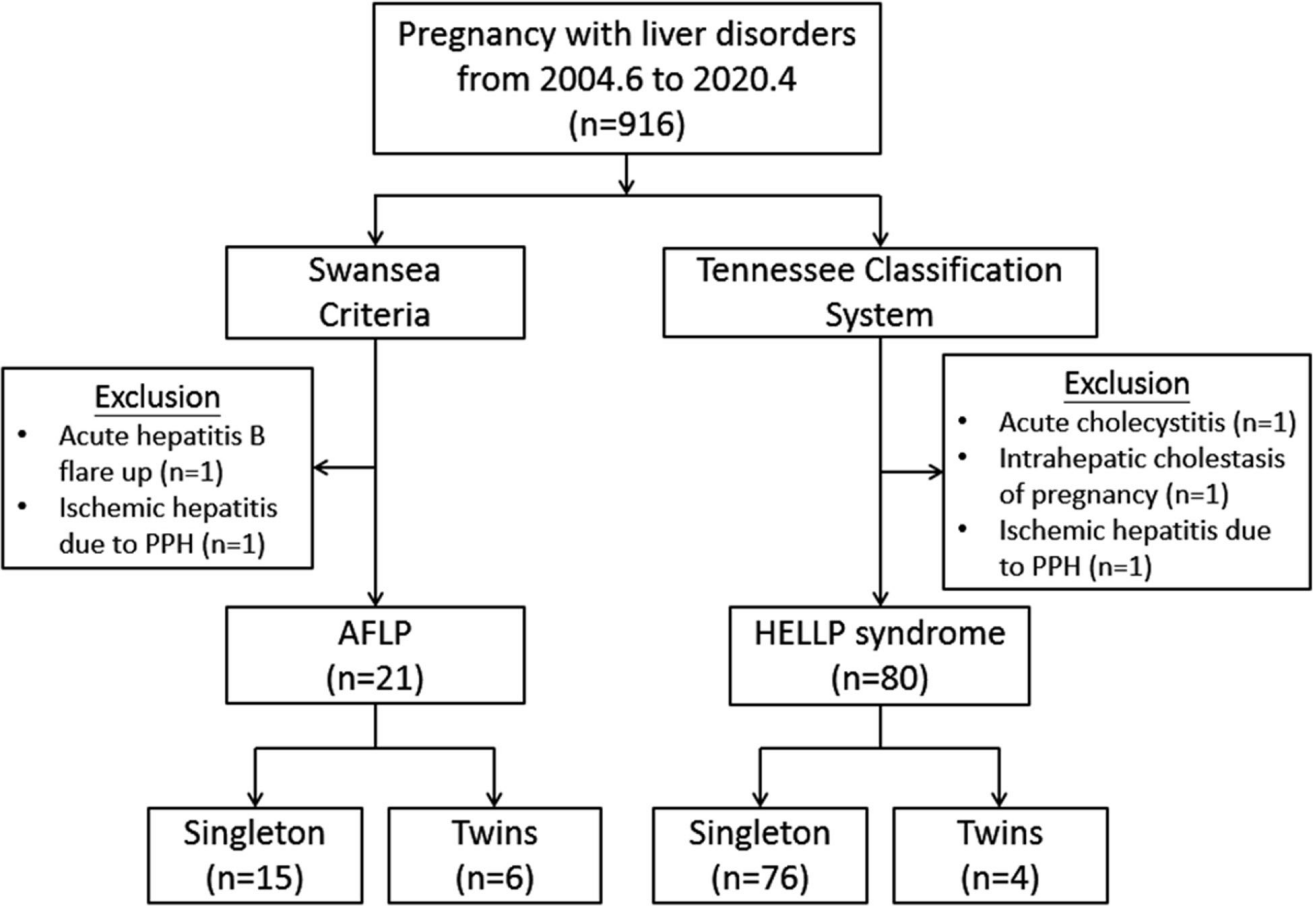
Flow diagram of the included women with acute fatty liver of pregnancy (AFLP) and hemolysis, elevated liver enzymes and low platelets (HELLP) syndrome. PPH: postpartum hemorrhage

During the study period, 916 pregnant women were initially diagnosed with liver disorders, of whom 106 pregnant women met the Swansea Criteria or the Tennessee Classification System. After excluding cases with acute hepatitis B flare up (*n* = 1), ischemic hepatitis due to postpartum hemorrhage (*n* = 2), acute cholecystitis (*n* = 1), and intrahepatic cholestasis of pregnancy (*n* = 1), 21 women were diagnosed with AFLP and 80 with HELLP syndrome (including 18 with complete and 62 with partial HELLP syndrome) (Fig. 1).

The maternal characteristics are shown in Table 2. The maternal body mass index (BMI) at delivery in the HELLP syndrome group (28.7 ± 5.1 [95 % confidence interval (CI) 27.5, 30] kg/m^2^) was significantly higher than that in the AFLP group (24.4 ± 3.3 [95 % CI 22.7, 26] kg/m^2^) (*P* = 0.001). Both maternal systolic and diastolic blood pressure levels on admission were significantly higher in the HELLP syndrome group than in the AFLP group (*P* < 0.001). The rate of twin pregnancy in the AFLP group was significantly higher than that in the HELLP syndrome group (28.6 % versus 5.0 %, *P* = 0.005). There were no significant differences in maternal age, gravidity, parity, and delivery age between the two groups. The frequency of cesarean delivery was also not significantly different between the AFLP and HELLP syndrome groups (90.5 % versus 85 %, *P* > 0.99).

**Table 2:** Maternal characteristics of the AFLP and HELLP syndrome groups

	AFLP (*n* = 21)	HELLP syndrome (*n* = 80)	*P*
Maternal age (years)^a^	33.5 ± 5.5 (31, 36)	33.7 ± 5.0 (32.5, 34.8)	0.920
Body mass index (kg/m^2^) at delivery^a^	24.4 ± 3.2 (22.7, 26)	28.7 ± 5.1 (27.5, 30)	0.001*
Gravida^b^	1.5 (1)	2 (2)	0.590
Para^b^	1 (1)	1 (1)	0.502
Nullipara^c^	15 (71.4)	50 (62.5)	0.447
Twin pregnancy^c^	6 (28.6)	4 (5.0)	0.005*
SBP on admission (mmHg)^a^	129.3 ± 14.6 (122.5, 136.1)	159.2 ± 21.5 (154.4, 164)	< 0.001*
DBP on admission (mmHg)^a^	75.1 ± 12.0 (69.5, 80.7)	96.4 ± 16.0 (92.9, 100)	< 0.001*
Chronic hypertension^c^	0 (0)	1 (1.3)	> 0.99
Gestational hypertension^c^	0 (0)	0 (0)	> 0.99
Delivery age (weeks)^b^	34.6 (3.4) (33, 36.3)	33.9 (6.9) (32.1, 34.9)	0.137
Cesarean delivery^c^	19 (90.5)	68 (85.0)	> 0.99

*AFLP* acute fatty liver of pregnancy; *HELLP* hemolysis, elevated liver enzymes and low platelets; *SBP* systolic blood pressure; *DBP* diastolic blood pressure

^a^Student’s *t* test, results are presented as mean ± standard deviation, (95 % confidence interval)

^b^Mann-Whitney U test, results are presented as median (interquartile range), (95 % confidence interval)

^c^Chi-square or Fisher’s exact test, results are presented as number (percentage)

*P < 0.05 was considered to be statistically significant

Table 3 shows the maternal laboratory data of the two groups. The results showed significant differences in medians or means for glucose (85.0 versus 98.5 mg/dL, *P* < 0.001), bilirubin (4.4 versus 0.9 mg/dL, *P* < 0.001), liver enzymes (AST: 257 versus 124 IU/L, *P* = 0.003; ALT: 316 versus 135 IU/L, *P* = 0.006), creatinine (1.8 versus 0.8 mg/dL, *P* < 0.001), and coagulation profile (prothrombin time: 14.1 versus 9.5 s, *P* < 0.001; activated partial thromboplastin time: 38.5 versus 27.9 s, *P* < 0.001; fibrinogen: 193 versus 408.1 mg/dL, *P* < 0.001), suggesting a higher frequency of hypoglycemia, hyperbilirubinemia, impaired liver function, impaired renal function, and coagulopathy in the AFLP group. However, the HELLP syndrome group had a significantly lower platelet count (89 × 10^3^/µL versus 174 × 10^3^/µL, *P* < 0.001) than the AFLP group. After adjusting by maternal BMI and twin pregnancy in a logistic regression analysis, there were no significant differences in AST, ALT, and creatinine levels between the two groups. No significant differences were noted in the levels of hemoglobin, leucocytes, uric acid, ammonia, and lactate dehydrogenase between the two groups. A further sub-analysis comparing the AFLP and complete HELLP syndrome groups revealed that there were still no significant differences in AST and ALT levels, but a higher LDH level in the complete HELLP syndrome group (765 versus 457 IU/L, *P* < 0.001) than the AFLP group (Table 3).

**Table 3:** Maternal laboratory data of the AFLP and HELLP syndrome groups

	AFLP versus HELLP syndrome					AFLP versus complete HELLP syndrome				
	AFLP (*n* = 21)	HELLP syndrome (*n* = 80)	*P*	*P*^*a*^	Adjusted B (95 % CI)	AFLP (*n* = 21)	Complete HELLP syndrome (*n* = 18)	*P*	*P*^*a*^	Adjusted B (95 % CI)
Hemoglobin (g/dL)^b^	13.4 ± 1.9 (12.5, 14.2)	12.4 ± 2.2 (11.9, 12.9)	0.069			13.4 ± 1.9 (12.5, 14.2)	12.6 ± 2.5 (11.3, 13.8)	0.272		
Leucocyte (10^6^/mL)^c^	14.5 (10.8) (9.9, 19.5)	11.8 (6.2) (10.7, 12.8)	0.362			14.5 (10.8) (9.9, 19.5)	12.7 (6.9) (10.5, 17)	0.662		
Platelet (10^3^/µL)^c^	174 (147) (130, 260)	89 (106.5) (76, 105)	< 0.001*	< 0.001*	97.37 (50.06, 144.68)	174 (147) (130, 260)	69.5 (41) (54, 91)	< 0.001*	< 0.001*	135.57 (74.41, 196.74)
Glucose (mg/dL)^c^	85 (29) (66, 95)	98.5 (2) (95, 104)	< 0.001*	0.001*	-19.80 (-31.30, -8.30)	85 (29) (66, 95)	97 (25) (94, 108)	0.004*	0.004*	-24.10 (-39.94, -8.27)
Bilirubin (mg/dL)^c^	4.4 (7.5) (2.4, 9.4)	0.9 (0.8) (0.8, 1.1)	< 0.001*	< 0.001*	5.65 (3.35, 7.95)	4.4 (7.5) (2.4, 9.4)	1.3 (1.5) (0.9, 2.4)	0.001*	0.040*	4.54 (0.23, 8.84)
AST (IU/L)^c^	257 (335) (153, 425)	124 (153) (93, 158)	0.003*	0.109	119.23 (-27.21, 265.66)	257 (335) (153, 425)	229 (375) (141, 496)	> 0.99		
ALT (IU/L)^c^	316 (389) (146, 520)	135 (138.8) (103, 165)	0.006*	0.074	162.14 (-16.28, 340.57)	316 (389) (146, 520)	181.5 (370) (94, 386)	0.345		
Uric acid (mg/dL)^c^	8.3 (2.1) (6.7, 9.7)	7.9 (1.4) (7.1, 9.1)	0.681			8.3 (2.1) (6.7, 9.7)	7.3 (2.3) (5.3, 11.7)	0.339		
Ammonia (µg/dL)^b^	79.9 ± 23.6 (67.3, 92.5)	66.0 ± 9.0 (56.5, 75.5)	0.154			79.9 ± 23.6 (67.3, 92.5)	67.5 ± 16.3 (56.3, 78.5)	0.488		
Creatinine (mg/dL)^c^	1.8 (1.1) (1.3, 2.4)	0.8 (0.5) (0.8, 1.1)	< 0.001*	0.059	0.51 (-0.02, 1.03)	1.8 (1.1) (1.3, 2.4)	0.9 (0.8) (0.7, 1.4)	0.005*	0.026*	0.60 (0.08, 1.13)
LDH (IU/L)^c^	457 (281) (282, 601)	489.5 (317.3) (402, 479)	0.535			457 (281) (282, 601)	765 (782) (641, 1346)	< 0.001*	0.041*	-342.51 (-670.38, -14.64)
PT (s)^c^	14.1 (10) (12.6, 19.9)	9.5 (1.3) (9.3, 9.7)	< 0.001*	< 0.001*	8.17 (4.71, 11.63)	14.1 (10) (12.6, 19.9)	9.6 (0.6) (9.4, 9.8)	< 0.001*	0.024*	8.38 (1.19, 15.57)
aPTT (s)^c^	38.5 (19.3) (32.5, 49.6)	27.9 (4.1) (27.5, 28.7)	< 0.001*	< 0.001*	16.34 (8.07, 24.62)	38.5 (19.3) (32.5, 49.6)	27.7 (3.1) (25.8, 28.4)	0.001*	0.043*	17.86 (0.62, 35.10)
Fibrinogen (mg/dL)^b^	193 ± 114.7 (126.7, 259.2)	408.1 ± 153.5 (366.2, 450)	< 0.001*	< 0.001*	-234.34 (-341.11, -127.57)	193 ± 114.7 (126.7, 259.2)	335.6 ± 121.4 (242.2, 428.9)	0.010*	0.006*	-218.82 (-364.01, 73.63)

*AFLP* acute fatty liver of pregnancy; *HELLP* hemolysis, elevated liver enzymes and low platelets; *CI* confidence interval; *AST* aspartate transaminase; *ALT* alanine aminotransferase; *LDH* lactate dehydrogenase; *PT* prothrombin time; *aPTT* activated partial thromboplastin time

^a^*P* adjusted by BMI and twin pregnancy status

^b^Student’s *t* test, results are presented as mean ± standard deviation, (95 % confidence interval)

^c^Mann-Whitney U test, results are presented as median (interquartile range), (95 % confidence interval)

*P < 0.05 was considered to be statistically significant

Table 4 shows the maternal complications of the two groups. There were significantly higher rates of preeclampsia (95 % versus 23.8 %, *P* < 0.001) and preeclampsia with severe features (95 % versus 4.8 %, *P* < 0.001) in the HELLP syndrome group compared to the AFLP group. Four patients in the HELLP syndrome group had severe features of preeclampsia but were normotensive, so they were not diagnosed as having preeclampsia. However, the AFLP group had more other complications including hypoglycemia (28.6 % versus 2.5 %, *P* = 0.001), jaundice (85.7 % versus 13.8 %, *P* < 0.001), AKI (61.9 % versus 15 %, *P* < 0.001), DIC (66.7 % versus 8.8 %, *P* < 0.001), and sepsis (47.6 % versus 10 %, *P* < 0.001) than the HELLP syndrome group. After adjusting by maternal BMI and twin pregnancy in a logistic regression analysis, we still revealed the similar significant differences. However, the further sub-analysis revealed that there were no differences in maternal complications of hypoglycemia, AKI, and sepsis between the AFLP and complete HELLP syndrome groups. There were no significant differences in rates of pulmonary edema, postpartum hemorrhage, gastrointestinal bleeding, wound hematoma, and placental abruption between the two groups. In addition, postpartum events, length of hospital stay, intensive care unit admission rate, need for blood transfusion or liver transplantation, and maternal mortality were not statistically different between the two groups. One patient with AFLP required a liver transplantation due to a rapid deterioration in liver function. In addition, one of the women died due to HELLP syndrome complicated by posterior reversible encephalopathy syndrome and intracranial hemorrhage.

**Table 4:** Maternal complications in the AFLP and HELLP syndrome groups

	AFLP versus HELLP syndrome					AFLP versus complete HELLP syndrome				
	AFLP (*n* = 21)	HELLP syndrome (*n* = 80)	*P*	*P*^*a*^	Adjusted OR (95 % CI)	AFLP (*n* = 21)	Complete HELLP syndrome (*n* = 18)	*P*	*P*^*a*^	Adjusted OR (95 % CI)
Preeclampsia^b^	5 (23.8)	76 (95.0)	< 0.001*	< 0.001*	0.02 (0.01, 0.11)	5 (23.8)	16 (88.9)	< 0.001*	0.006*	0.03 (0.01, 0.35)
Preeclampsia with severe features^b^	1 (4.8)	76 (95.0)	< 0.001*	< 0.001*	0.01 (0.01, 0.03)	1 (4.8)	16 (88.9)	< 0.001*	0.002*	0.01 (0.01, 0.13)
Hypoglycemia^b^	6 (28.6)	2 (2.5)	0.001*	0.026*	11.28 (1.33, 95.66)	6 (28.6) (12.8, 52.1)	1 (5.6) (0.7, 33.4)	0.098		
Jaundice^b^	18 (85.7)	11 (13.8)	< 0.001*	< 0.001*	32.45 (6.12, 171.94)	18 (85.7)	5 (27.8)	< 0.001*	0.007*	14.94 (2.08, 107.29)
Pulmonary edema^b^	2 (9.5)	8 (10.0)	> 0.99			2 (9.5)	5 (27.8)	0.215		
AKI^b^	13 (61.9)	12 (15.0)	< 0.001*	0.003*	6.78 (1.89, 24.32)	13 (61.9)	6 (33.3)	0.075		
DIC^b^	14 (66.7)	7 (8.8)	< 0.001*	< 0.001*	12.67 (3.11, 51.56)	14 (66.7)	1 (5.6)	< 0.001*	0.012*	21.27 (1.95, 231.64)
PPH^b^	2 (9.5)	10 (12.5)	> 0.99			2 (9.5)	4 (22.2)	0.387		
Sepsis^b^	10 (47.6)	8 (10.0)	< 0.001*	0.036*	4.30 (1.10, 16.83)	10 (47.6)	4 (22.2)	0.099		
Gastrointestinal bleeding^b^	1 (4.8)	0 (0)	0.208			1 (4.8)	0 (0)	> 0.99		
Wound hematoma^b^	2 (9.5)	2 (2.5)	0.190			2 (9.5)	1 (5.6)	> 0.99		
Placental abruption^b^	1 (4.8)	5 (6.3)	> 0.99			1 (4.8)	0 (0)	> 0.99		
Blood transfusion^b^	8 (38.1)	17 (21.3)	0.111			8 (38.1)	6 (33.3)	0.757		
Liver transplantation^b^	1 (4.8)	0 (0)	0.208			1 (4.8)	0 (0)	> 0.99		
Postpartum event^b^	1 (4.8)	10 (12.5)	0.451			1 (4.8)	4 (22.2)	0.162		
Length of hospitalization (days)^c^	9 (7.0)	6 (5.8)	0.066			9 (7.0)	6.5 (7.0)	0.223		
ICU admission^b^	4 (19.0)	8 (10.0)	0.267			4 (19.0)	3 (16.7)	> 0.99		
Maternal death^b^	0 (0)	1 (1.3)	> 0.99			0 (0)	1 (5.6)	0.462		

*AFLP* acute fatty liver of pregnancy; *HELLP* hemolysis, elevated liver enzymes and low platelets; *OR* odds ratio; *CI* confidence interval; *AKI* acute kidney injury; *DIC* disseminated intravascular coagulopathy; *PPH* postpartum hemorrhage; *ICU* intensive care unit

^a^*P* adjusted by BMI and twin pregnancy

^b^Chi-square or Fisher’s exact test, results are presented as number (percentage)

^c^Mann-Whitney U test, results are presented as median (interquartile range)

*P < 0.05 was considered to be statistically significant

The neonatal outcomes are shown in Table 5. The birth weight of the HELLP syndrome group was significantly lower than that of the AFLP group (1686.1 ± 735.1 [95 % CI 1518.1, 1854] versus 2235.5 ± 591.7 [95 % CI 1996.5, 2474.5] g, *P* = 0.001). The rate of SGA neonates was significantly higher in the HELLP syndrome group (57.1 % versus 33.3 %, *P* = 0.031). In addition, higher rates of RDS (39.2 % versus 8.3 %, *P* = 0.004) and sepsis (34.2 % versus 12.5 %, *P* = 0.041) were noted in the HELLP syndrome group. There were no significant differences in birth age, Apgar scores, sex, stillbirth, intracranial hemorrhage, transient tachypnea of the newborn, persistent pulmonary hypertension of the newborn, neonatal intensive care unit admission rate, and neonatal mortality between the two groups. Six neonates died in the HELLP syndrome group, of whom five were due to prematurity, and one was due to sepsis. A logistic regression analysis adjusting by maternal BMI and twin pregnancy still revealed the similar significant differences. A further sub-analysis comparing the AFLP and complete HELLP syndrome groups also revealed the similar results (Table 5).

**Table 5:** Neonatal outcomes of the AFLP and HELLP syndrome groups

	AFLP versus HELLP syndrome					AFLP versus complete HELLP syndrome				
	AFLP (*n* = 27)	HELLP syndrome (*n* = 84)	*P*	*P*^*a*^	Adjusted B or OR (95 % CI)	AFLP (*n* = 27)	Complete HELLP syndrome (*n* = 18)	*P*	*P*^*a*^	Adjusted B or OR (95 % CI)
Birth age (weeks)^b^	34.6 (3.4) (33, 36)	33.9 (6.9) (32, 34)	0.137			34.6 (3.4) (33, 36)	34 (4) (32, 36)	0.449		
Birth weight (g)^c^	2235.5 ± 591.7 (1996.5, 2474.5)	1686.1 ± 735.1 (1518.1, 1854)	0.001*	0.006*	590.5 (177.7, 1003.4)	2235.5 ± 591.7 (1996.5, 2474.5)	1784.0 ± 583.17 (1473.3, 2094.7)	0.020*	0.015*	606.97 (126.55, 1087.38)
Apgar score (1 min)^b^	8 (5)	7 (3)	0.540			8 (5)	6.5 (2)	0.378		
Apgar score (5 min)^b^	9 (3)	9 (1)	0.512			9 (3)	8.5 (1)	0.328		
Male^d^	17/27 (63)	45/84 (53)	0.393			17/27 (63)	12/18 (66.7)	0.799		
SGA^d^	9/27 (33.3)	48/84 (57.1)	0.031*	0.049*	0.32 (0.10, 0.99)	9/27 (33.3)	12/18 (66.7)	0.028*	0.029*	0.15 (0.03, 0.82)
Stillbirth^d^	3/27 (11.1)	5/84 (6)	0.400			3/27 (11.1)	0/18 (0)	0.143		
Live birth complication										
ICH^d^	0/24 (0)	3/79 (3.8)	> 0.99			0/24 (0)	0/18 (0)	NA		
RDS^d^	2/24 (8.3)	31/79 (39.2)	0.004*	0.012*	0.11 (0.02, 0.61)	2/24 (8.3)	7/18 (38.9)	0.025*	0.042*	0.14 (0.02, 0.93)
TTN^d^	11/24 (45.8)	31/79 (39.2)	0.565			11/24 (45.8)	11/18 (61.1)	0.327		
PPHN^d^	0/24 (0)	2/79 (2.5)	> 0.99			0/24 (0)	0/18 (0)	NA		
Sepsis^d^	3/24 (12.5)	27/79 (34.2)	0.041*	0.024*	0.17 (0.04, 0.79)	3/24 (12.5)	8/18 (44.4)	0.033*	0.033*	0.12 (0.02, 0.85)
NICU admission^d^	10/24 (41.7)	43/79 (54.4)	0.273			10/24 (41.7)	11/18 (61.1)	0.212		
Neonatal mortality^d^	0/24 (0)	6/79 (7.6)	0.332			0/24 (0)	0/18 (0)	NA		

*AFLP* acute fatty liver of pregnancy; *HELLP* hemolysis, elevated liver enzymes and low platelets; *OR* odds ratio; *CI* confidence interval; *SGA* small for gestational age; *ICH* intracranial hemorrhage; *NA* non-applicable; *RDS* respiratory distress syndrome; *TTN* transient tachypnea of the newborn; *PPHN* persistent pulmonary hypertension of the newborn; *NICU* neonatal intensive care unit

^a^*P* adjusted by maternal body mass index and twin pregnancy

^b^Mann-Whitney U test, results are presented as median (interquartile range), (95 % confidence interval)

^c^Student’s t test, results are presented as mean ± standard deviation, (95 % confidence interval)

^d^Chi-square or Fisher’s exact test, results are presented as number (percentage)

*P < 0.05 was considered to be statistically significant

**Fig. 2:**
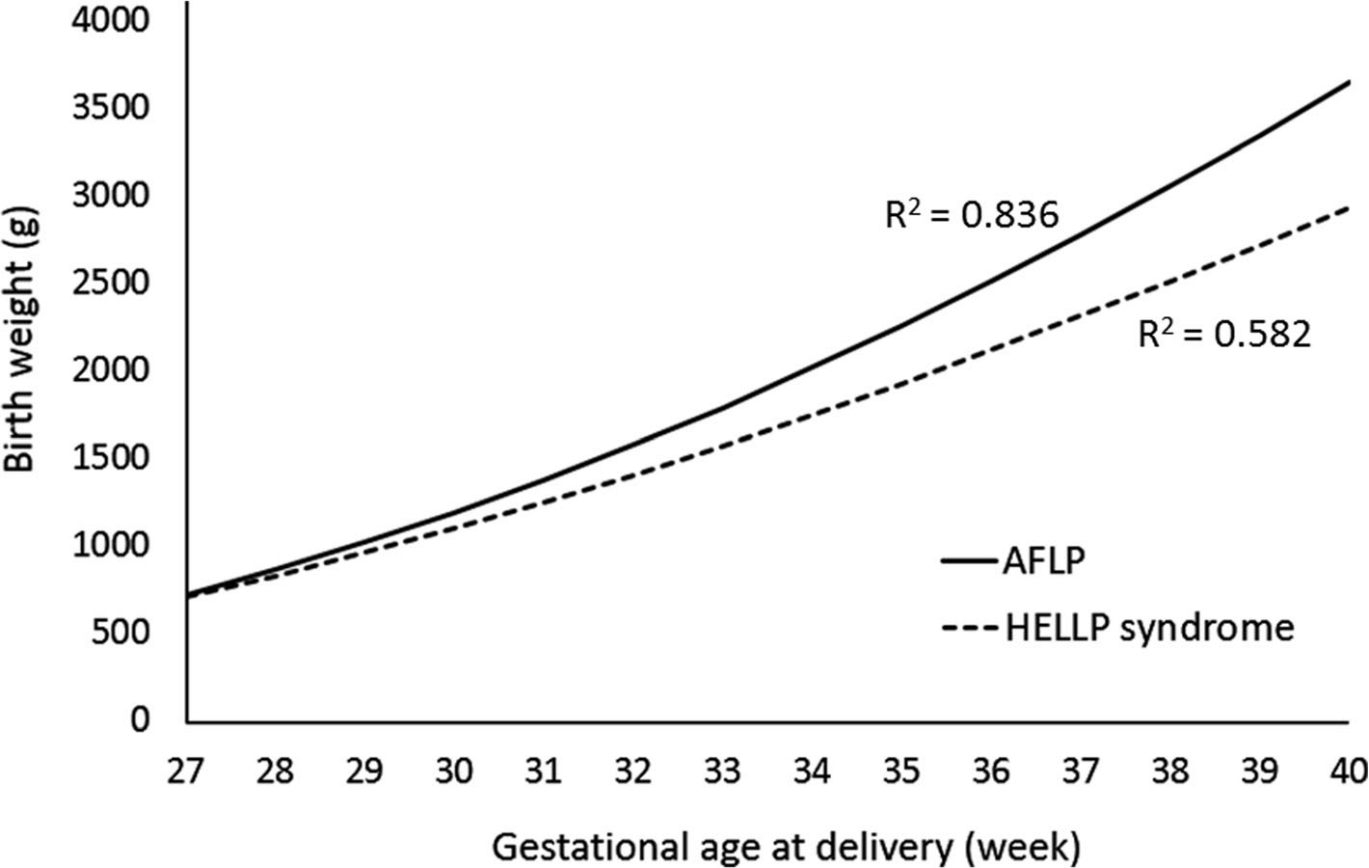
S-curve regression analysis of birth weight against gestational age at delivery between the acute fatty liver of pregnancy (AFLP) and hemolysis, elevated liver enzymes and low platelets (HELLP) syndrome groups. The coefficients of determination (*R*^2^) for AFLP and HELLP syndrome were 0.836 and 0.582, respectively

An S-curve regression model was used to analyze birth weight against gestational age at delivery between the AFLP and HELLP syndrome groups, and the coefficients of determination (*R*^2^) were 0.836 and 0.582, respectively (Fig. 2). It showed a gradual diverging trend of the two curves towards a higher birth weight in the AFLP group and lower birth weight in the HELLP syndrome group.

## Discussion

In this study, the women with AFLP were associated with jaundice and DIC, whereas those with HELLP syndrome were associated with hypertension on admission, preeclampsia and low platelet counts. We also demonstrated a significantly higher rate of twin pregnancy in the AFLP group. However, more neonatal complications were noted in the HELLP syndrome group, such as SGA, RDS, and neonatal sepsis. Accordingly, clinical and laboratory evidence of hyperbilirubinemia, hypofibrinogenemia, and coagulopathy may help to differentiate AFLP from HELLP syndrome.

AFLP and HELLP syndrome differed in the magnitude of systemic involvement. Multiple organ dysfunction was more likely to occur in the patients with AFLP. Hypoglycemia results from impaired hepatic glycogenolysis, and AFLP with hepatic steatosis can inhibit bilirubin clearance resulting in hyperbilirubinemia and jaundice [23]. AKI can be caused by defective renal fatty acid oxidation, and fatty degeneration in renal tubules [24] while severe hepatic impairment can also lead to hepatorenal syndrome. The major cause of coagulopathy resulting in DIC is the reduction in hepatic production of fibrinogen and other procoagulant proteins [23]. Compared to the women with AFLP, those with HELLP syndrome had a higher BMI at delivery, which is consistent with previous studies that obesity is one of the risk factors of HELLP syndrome [25].

Even with the various maternal complications associated with AFLP, neonatal morbidities in the AFLP group were not as high as those in the HELLP syndrome group. This may be because the toxic unoxidized fatty acids due to LCHAD deficiency in the AFLP group were transferred to the mother through the placenta instead of accumulating in the fetus, thereby not increasing neonatal morbidities compared to the HELLP syndrome group.

As would be anticipated, HELLP syndrome was more likely to occur in the patients with preeclampsia and a low platelet count. Although the relationship between HELLP syndrome and preeclampsia is still controversial, HELLP syndrome is usually considered to be a severe form or a variant of preeclampsia [16]. In addition, HELLP syndrome is related to endothelial injury and microangiopathic platelet consumption, which results in thrombocytopenia [11].

Twin pregnancy has been reported to be a risk factor for both AFLP and HELLP syndrome [13]. However, in this head-to-head comparison study, the rate of twin pregnancy in the AFLP group was significantly higher than that in the HELLP syndrome group. Most previous studies have indicated that fetal LCHAD deficiency [8] and other types of deficiencies in fetoplacental mitochondrial oxidation, such as short-chain acyl-coenzyme dehydrogenase deficiency and carnitine palmitoyltransferase deficiency are related to the development of AFLP [26, 27]. Accordingly, it has been hypothesized that twice the amount of upstream metabolites accumulate in the circulation of mothers with twins due to these enzyme deficiencies, resulting in a lower threshold to express clinical symptoms and signs in patients with AFLP.

Another important finding of this study is the significantly higher rate of SGA neonates born to the mothers with HELLP syndrome. Although twin pregnancy is known to be a key factor leading to SGA, we found a higher rate of twin pregnancy in the AFLP group rather than the HELLP syndrome group. This could be explained by the abnormal placentation in patients with HELLP syndrome. Alterations in platelet activation, increases in pro-inflammatory cytokines, and segmental vasospasm with vascular endothelial damage could impair nutritional exchange through the feto-placental unit and subsequently result in SGA [4, 8]. In addition, higher rates of neonatal RDS and sepsis were also noted in the HELLP syndrome group. A correlation between SGA and RDS has been reported, possibly because intrauterine lung development can be adversely affected by fetal growth restriction due to reduced substrate supply, fetal hypoxemia and hypercortisolemia [28]. The possible mechanism of increased sepsis in SGA neonates is the delayed immune system development, which results in a higher rate of neonatal infection [29].

The strengths of this study include that this was a head-to-head comparison study of both maternal and neonatal outcomes. Moreover, the data were collected from one medical center, which could minimize management bias in different institutions. Nevertheless, there are several limitations to this study. First, the data were collected from one medical center, and thus the case numbers were limited even a near 17-year span. Second, advances in maternal and neonatal care in the recent two decades may have resulted in different maternal and neonatal outcomes. Third, in this retrospective study we did not compare the two pregnancy-related liver disorders with a reference group due to the limited laboratory data of biochemical and coagulation profile in patients without the two diseases. A further prospective case-control study to compare women with AFLP, HELLP syndrome, and no hepatic disease can provide more clarity on this issue.

## Conclusions

Both AFLP and HELLP syndrome affect liver function yet differ in the magnitude of systemic involvement. Multiple organ dysfunction was more likely to occur in the patients with AFLP. However, more neonatal morbidities were noted in the HELLP syndrome group. These results may help us to differentiate between the two pregnancy-related liver disorders, and when explaining the management and prognosis during shared decision making with patients.

## Data Availability

Data have been collected at Mackay Memorial Hospital, a tertiary referral hospital in Taiwan. The classified and organized databases as the source of data for this study can be found at the study data registry of this hospital.

## Abbreviations

IRB: Institutional Review Board
AFLP: Acute fatty liver of pregnancy
HELLP: Hemolysis, elevated liver enzymes and low platelets
LCHAD: Long-chain 3-hydroxyacyl coenzyme A dehydrogenase
AST: Aspartate transaminase
ALT: Alanine aminotransferase
AKI: Acute kidney injury
DIC: Disseminated intravascular coagulopathy
SGA: Small for gestational age
RDS: Respiratory distress syndrome
BMI: Body mass index
CI: Confidence interval
